# Did saber-tooth kittens grow up musclebound? A study of postnatal limb bone allometry in felids from the Pleistocene of Rancho La Brea

**DOI:** 10.1371/journal.pone.0183175

**Published:** 2017-09-27

**Authors:** Katherine Long, Donald Prothero, Meena Madan, Valerie J. P. Syverson

**Affiliations:** 1 Department of Geological Sciences, California State Polytechnic University, Pomona, California, United States of America; 2 Department of Vertebrate Paleontology, Natural History Museum of Los Angeles County, Los Angeles, California, United States of America; 3 School of Earth Sciences, University of Bristol, Bristol, United Kingdom; 4 Department of Geological Sciences, University of Wisconsin, Madison, Wisconsin, United States of America; Royal Belgian Institute of Natural Sciences, BELGIUM

## Abstract

Previous studies have demonstrated that the Pleistocene saber-toothed cat *Smilodon fatalis* had many forelimb adaptations for increased strength, presumably to grapple with and subdue prey. The Rancho La Brea tar pits yield large samples of juvenile limb bones forming a growth series that allow us to examine how *Smilodon* kittens grew up. Almost all available juvenile limb bones were measured, and reduced major axis fits were calculated to determine the allometric growth trends. Contrary to expectations based on their robust limbs, *Smilodon* kittens show the typical pattern of growth found in other large felids (such as the Ice Age lion, *Panthera atrox*, as well as living tigers, cougars, servals, and wildcats) where the limb grows longer and more slender faster than they grow thick. This adaptation is thought to give felids greater running speed. *Smilodon* kittens do not grow increasingly more robust with age. Instead, they start out robust and follow the ancestral felid growth pattern, while maintaining their robustness compared to other felids. Apparently, the growth of felid forelimbs is highly canalized and their ontogeny is tightly constrained.

## Introduction

**Fig 1:**
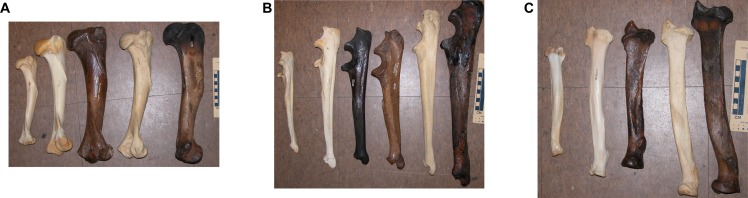
Visual comparison of the forelimbs of large felids all shot in the same frame for scale, showing the relative robustness of *Smilodon* compared to cats of similar size. The light-colored bone on the left end is the cougar, *Puma concolor*; the light-colored bone second from left is the tiger, *Panthera tigris*; the dark bone(s) in the middle are *Smilodon fatalis*; the light-colored bone second from right is the lion, *Panthera leo*; the dark bone on the right is the Ice Age lion, *Panthera atrox*;. Scale bar in cm. A. Humerus. B. Ulna. C. Radius. Photos by DRP.

For decades, paleontologists have noted that saber-toothed cats, such as the North American Pleistocene *Smilodon fatalis*, are more robustly built (Fig 1) than most felids [1–10]. Most recent authors have concluded that saber-toothed predators probably killed their prey with quick slashing bites to the throat, using powerful forelimbs to hold the prey down during attack [2–10]. Meachen-Samuels and Van Valkenburgh [9] used X-radiographs to show that *Smilodon* forelimbs were more robust and had thicker cortical bone than comparable bones of most other similar-sized felids.

**Fig 2:**
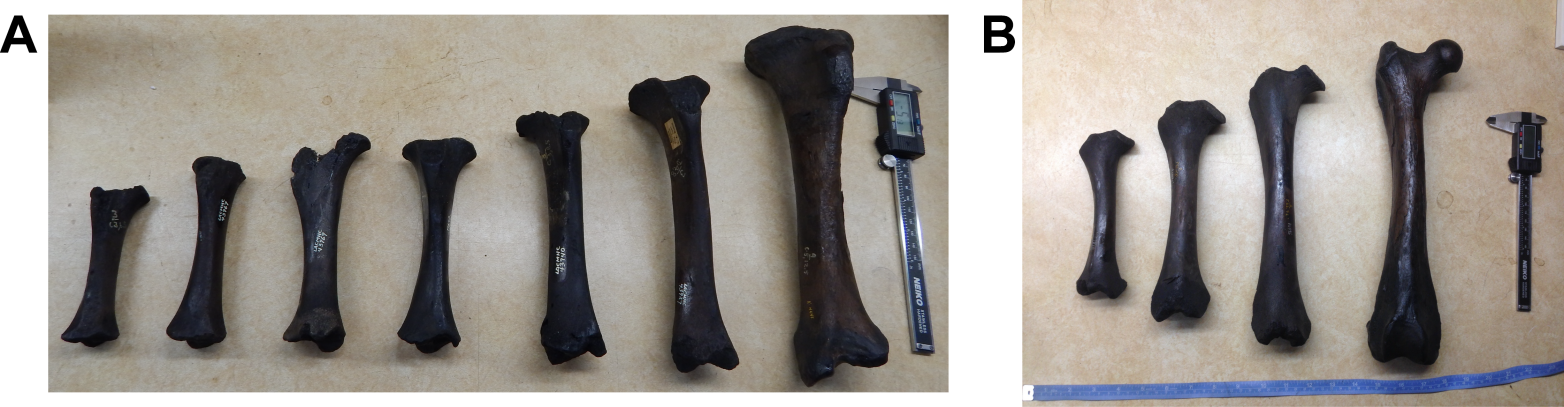
Growth series of juvenile limb bones of *Smilodon fatalis*, showing the dramatic transformation in size and shape during ontogeny. A. Tibia. B. Femora. Photos by DRP.

But how and when did *Smilodon* kittens acquire their robust forelimbs as they grew? Do they have a more robust growth pattern than other felids, or do they achieve this robustness by other means? Very few fossil mammals are known from enough juvenile limb bones to assess this question. However, the collections of late Pleistocene fossils from Rancho La Brea tar pits yield thousands of bones of *Smilodon fatalis* and the contemporary tiger-sized felid *Panthera atrox*, including hundreds of juvenile bones in various ontogenetic stages (Fig 2). These are among the few fossil collections in the world that allow research in postnatal ontogenetic allometry.

Until recently, there was very little published literature on postnatal ontogenetic allometry of living mammals to allow comparison with the fossil samples at Rancho La Brea. Kilbourne and Makovicky [11] provided the first published data on postnatal long-bone growth in several living felids, including the tiger (*Panthera tigris*), the cougar (*Puma concolor*), the Old World wildcat (*Felis silvestris*), and the serval (*Leptailurus serval*). Their data and analyses provide a standard to which we can compare juvenile bones of La Brea felids.

Kilbourne and Makovicky [11] found that many living felids show evidence of more rapid growth of the length of their distal limb elements, relative to their cross-sectional area, so their limbs become more gracile as they grow. This is a common adaptation of many cursorial mammals, which have lengthened their distal limb elements (radius, ulna, tibia) relative to bone shaft thickness. Biomechanical studies have shown that this decreases the rotational inertia in longer-limbed animals [12, 13]. In this study, we compare the tiger-sized *Panthera atrox* to determine whether it grew to its adult body proportions in the same way as do modern tiger kittens. We also wanted to see if *Smilodon fatalis* kittens grew up with the same changes in their limb proportions as other felids, or with a more robust ontogenetic limb growth pattern than other felids.

## Materials and methods

We measured nearly every unbroken juvenile humerus, radius, femur, and tibia of *S*. *fatalis* and *P*. *atrox* in the collections of the La Brea Tar Pits Museum (formerly the George C. Page Museum) to get the largest sample size possible for this analysis. These specimens have a variety of different catalogue numbering systems, but all come from the main curated collection in the La Brea Tar Pits Museum. Catalogue numbers of all specimens are given in the original data tables (S1 Table, S2 Table). Sample sizes are given in Tables 1–8; original data are given in the Supplementary Tables. Measurements were made following the protocols of Kilbourne and Makovicky [11], focusing on measuring the length of the diaphysis of the shaft of each bone. In the case of adult or subadult bones, measurements of shaft length were made from the diaphyseal-epiphyseal sutures. Measurements shorter than 460 mm were made with metric digital calipers; those over 460 mm, and circumference measurements, were made with a flexible metric measuring tape. In addition to measuring diaphysis length, we measured midshaft circumference, and two linear measures of the midshaft: lateral width and antero-posterior depth. This allowed us to compare diaphysis lengths to both circumference, and also cross-sectional area.

**Table 1:** Humerus data of various mammals.

TAXON	N	L.S. Slope	Y-INTERCEPT	R^2^	SLOPE C.I.	RMA
*Smilodon fatalis*	30	1.389	-1.1781	0.885	1.46, 1.85	1.65 (G)
*Panthera atrox*	14	1.208	-0.2912	0.831	1.02, 1.71	1.32 (G)
*Panthera tigris*	13	1.269	-0.2093	0.962	1.13, 1.47	1.29 (G)
*Puma concolor*	15	0.794	1.9882	0.838	0.68, 1.10	0.87 (I)
*Felis sylvestris*	35	1.477	-0.2271	0.960	1.41, 1.62	1.51 (G)
*Leptailurus serval*	15	1.354	-0.1812	0.906	1.18, 1.71	1.42 (G)

L.S. SLOPE = least squares slope; SLOPE C.I. = slope confidence interval; RMA = reduced major axis slope. The results of the RMA of the slopes are coded as follows, using the criteria of Kilbourne and Makovicky [11]: (G) = gracile; (I) = isometric; (R) = robust. *S*. *fatalis* and *P*. *atrox* results from this study; all other data from [11]. (No least squares slopes were given by [11]).

**Table 2:** Humerus data of various mammals. Conventions as in Table 1.

TAXON	N	L.S. Slope	Y-INTERCEPT	R^2^	SLOPE C.I.	RMA
*Smilodon fatalis*	30	0.6913	0.5188	0.8819	0.66, 0.85	0.76 (R)
*Panthera atrox*	15	0.5814	1.3233	0.8867	0.50, 0.76	0.62 (R)

**Table 3:** Radius data of various mammals. Conventions as in Table 1.

TAXON	N	L.S. Slope	Y-INTERCEPT	R^2^	SLOPE C.I.	RMA
*Smilodon fatalis*	35	1.3918	-0.8086	0.932	1.31, 1.58	1.44 (G)
*Panthera atrox*	8	0.7302	2.2884	0.906	0.27, 0.43	0.34 (R)
*Panthera tigris*	12	1.316	0.0371	0.986	1.22, 1.44	1.33 (G)
*Puma concolor*	14	0.853	1.8600	0.902	0.74, 1.09	0.90 (I)
*Felis sylvestris*	25	1.294	0.7792	0.88	1.19, 1.60	1.38 (G)

**Table 4:** Radius data of various mammals. Conventions as in Table 1.

TAXON	N	L.S. Slope	Y-INTERCEPT	R^2^	SLOPE C.I.	RMA
*Smilodon fatalis*	35	0.6243	0.4795	0.9392	0.51, 0.80	0.64 (R)
*Panthera atrox*	8	0.5814	1.3233	0.9467	0.27, 0.43	0.34 (R)

**Table 5:** Femoral data of various mammals. Conventions as in Table 1.

TAXON	N	L.S. Slope	Y-INTERCEPT	R^2^	SLOPE C.I.	RMA
*Smilodon fatalis*	30	1.009	1.2882	0.904	1.46, 1.85	1.65 (G)
*Panthera atrox*	8	1.392	-0.8612	0.733	0.99, 2.66	1.63 (G)
*Panthera tigris*	15	1.453	-0.679	0.985	1.36, 1.58	1.46 (G)
*Puma concolor*	14	1.004	1.004	0.766	0.85, 1.54	1.15 (I)
*Felis sylvestris*	36	1.572	1.382	0.961	1.50, 1.72	1.61 (G)
*Leptailurus serval*	15	1.396	-0.1511	0.974	1.29, 1.56	1.41 (G)

**Table 6:** Femoral data of various mammals. Conventions as in Table 1.

TAXON	N	L.S. Slope	Y-INTERCEPT	R^2^	SLOPE C.I.	RMA
*Smilodon fatalis*	30	0.4739	-0.4255	0.893	0.66, 0.85	0.76 (R)
*Panthera atrox*	8	0.7028	0.7794	0.823	0.51, 1.16	0.77 (R)

**Table 7:** Tibia data of various mammals. Conventions as in Table 1.

TAXON	N	L.S. Slope	Y-INTERCEPT	R^2^	SLOPE C.I.	RMA
*Smilodon fatalis*	70	1.524	-0.4087	0.7927	1.53, 1.91	1.71 (G)
*Panthera atrox*	24	1.185	0.4381	0.7905	0.88, 1.32	1.08 (I)
*Panthera tigris*	12	1.433	-0.6792	0.980	1.31, 1.60	1.45 (G)
*Puma concolor*	14	0.770	2.240	0.752	0.65, 1.21	0.89 (I)
*Felis sylvestris*	30	1.499	-0.1502	0.936	1.40, 1.71	1.55 (G)
*Leptailurus serval*	12	1.208	0.8269	0.976	1.10, 1.36	1.22 (G)

**Table 8:** Tibia data of various mammals. Conventions as in Table 1.

TAXON	N	L.S. Slope	Y-INTERCEPT	R^2^	SLOPE C.I.	RMA
*Smilodon fatalis*	70	0.4592	1.617	0.619	0.32, 0.49	0.40 (R)
*Panthera atrox*	24	0.6013	1.835	0.8303	0.43, 0.66	0.54 (R)

Basic statistics and regressions were calculated and plotted using Microsoft Excel. Although they do not state it clearly, Kilbourne and Makovicky [11] plotted length on the ordinate and circumference on the abscissa, so that convention is followed here as well. Following the conventions of allometric studies, raw data were log-transformed using natural logs, and plotted in log-log space, so that the exponential slope of allometry would give a simple linear slope. We used Excel to calculate the simple least-squares regression of the data. Since there is no dependent or independent variable in this study, as the least-squares regression method assumes, we adopted the more commonly used Reduced Major Axis (RMA) method of correlation to determine the slope between the two variables (calculated using the “smatr” software routine for the R software package) (S3 Table). This software package also calculates the slope confidence interval (CI). However, we found there were slight differences between our RMA results and those published by Kilbourne and Makovicky [11], even when we analyzed their original data with the R software package. Consequently, in Tables 1, 3, 5 and 7, we calculated all our own results, rather than reprint the numbers given by Kilbourne and Makovicky [11].

We followed the conventions of Kilbourne and Makovicky [11] in plotting length vs. circumference, which should give an isometric slope in log-log space of approximately 1.0 (linear dimension vs. linear dimension). This allowed us to compare our results to the data of living mammals examined by Kilbourne and Makovicky [11]. We also calculated cross-sectional area and compared it to shaft length, to see if it scaled along the expected isometric slope of 2.0 (length vs. area), or if it was significantly allometric with a slope very different from 2.0. Supporting information is available elsewhere (S1 Table and S2 Table)

## Results

### Length vs. circumference

**Fig 3:**
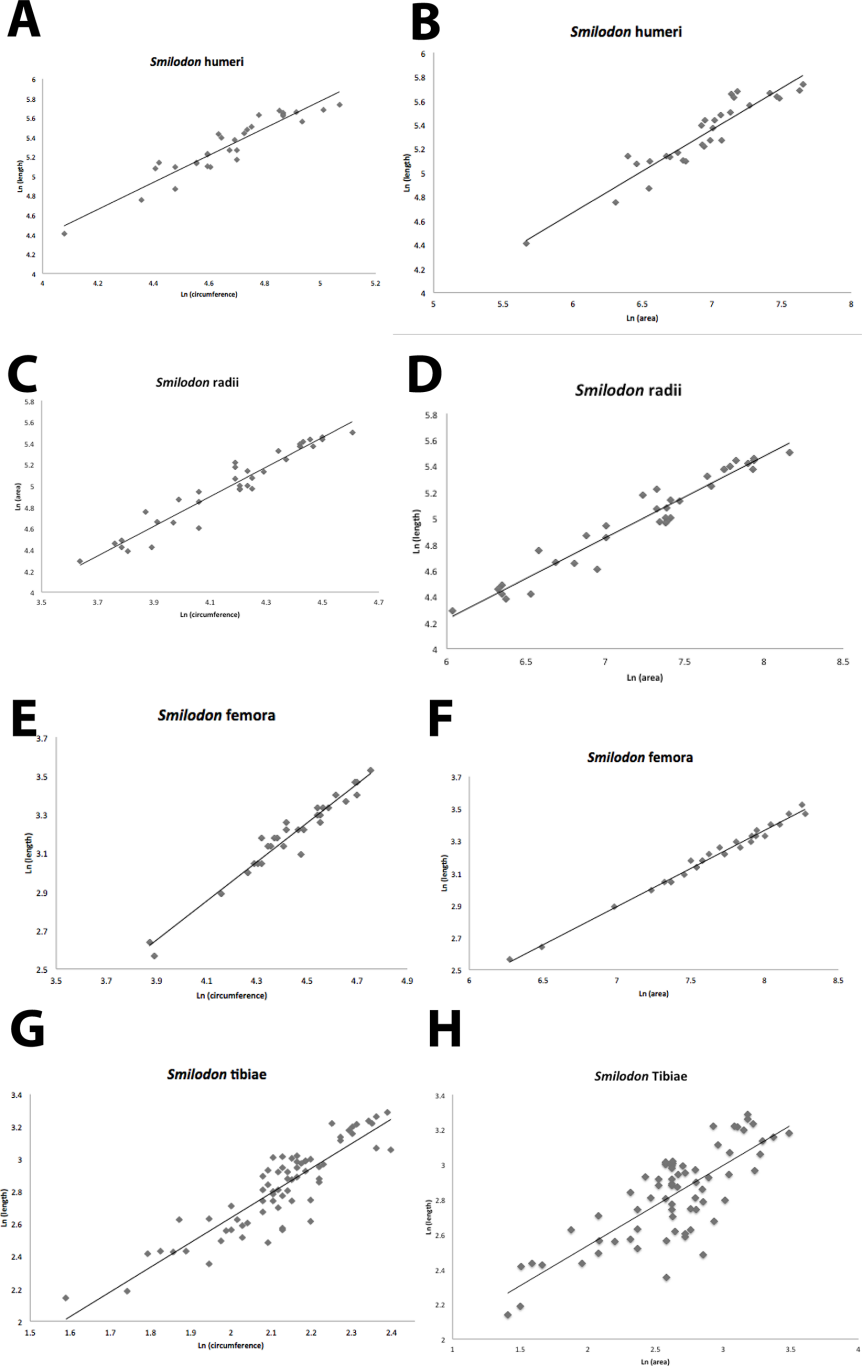
Bivariate plots of data of limb bone length vs. circumference for *Smilodon fatalis* and *Panthera atrox*. A-B. Humerus length vs. circumference for *Smilodon* (A) and *P*. *atrox* (B). C-D. Radius length vs. circumference for *Smilodon* (C) and *P*. *atrox* (D). E-F. Femur length vs. circumference for for *Smilodon* (E) and *P*. *atrox* (F).G-H. Tibia length vs. circumference for *Smilodon* (G) and *P*. *atrox* (H).

**Fig 4:**
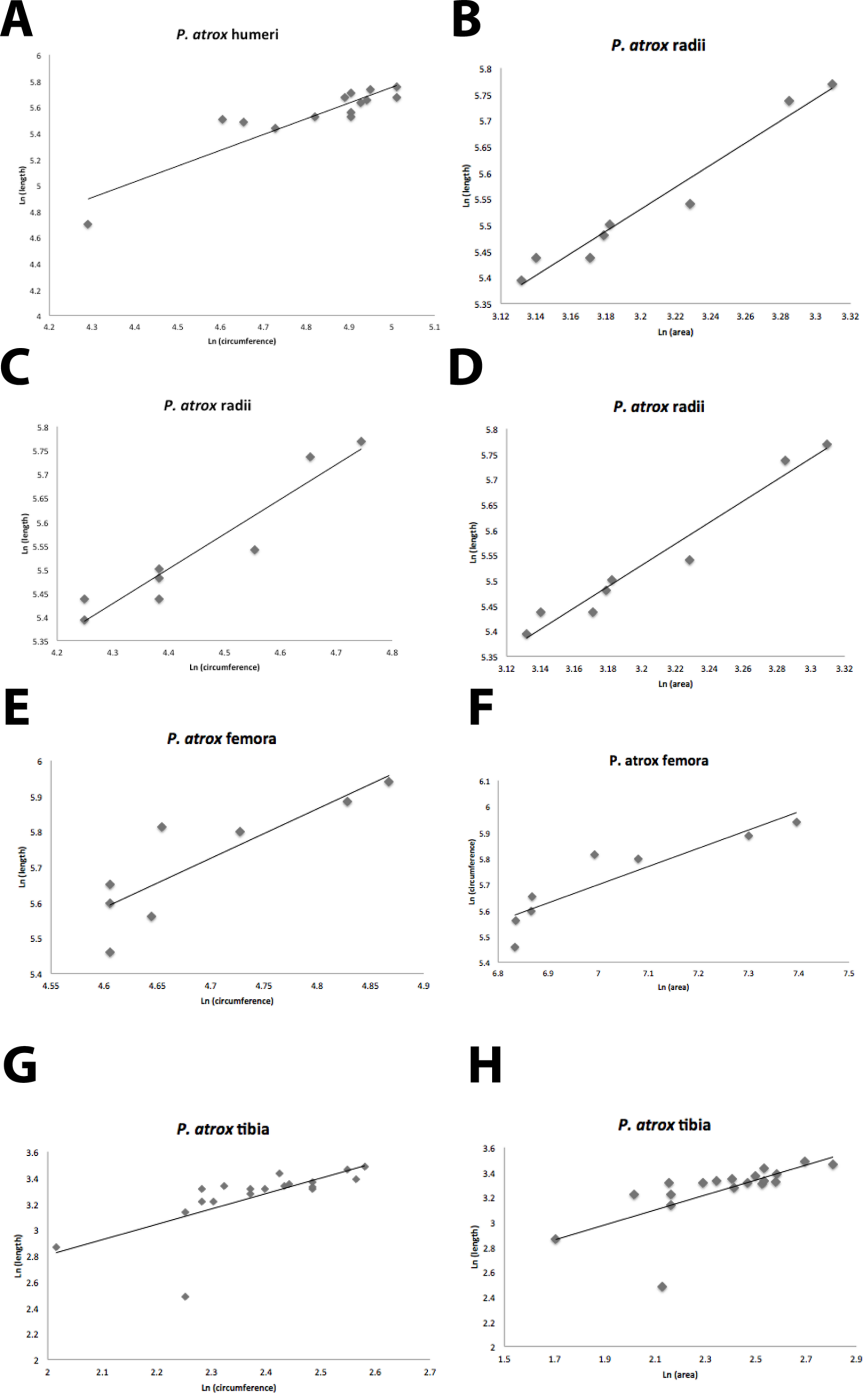
Bivariate plot of data of limb bone length vs. area for *Smilodon fatalis* and *Panthera atrox*. A-B. Humerus length vs. area for *Smilodon* (A) and *P*. *atrox* (B). C-D. Radius length vs. area for *Smilodon* (C) and *P*. *atrox* (D). E-F. Femur length vs. area for *Smilodon* (E) and *P*. *atrox* (F). G-H. Tibia length vs. area for *Smilodon* (G) and *P*. *atrox* (H).

Our results are plotted in Figs 3 and 4, and reported in Tables 1, 3, 5 and 7. Using a least-squares regression analysis first, we found very high correlation coefficients (R^2^) ranging from 0.793 to 0.932 in *S*. *fatalis*, which had larger sample sizes, and from 0.733 to 0.906 for *P*. *atrox*, which had fewer specimens. Least-squares regression slopes ranged from 1.0–1.5 in *S*. *fatalis*, suggesting that all its limb bones became more robust as it grew, and from 0.7 to 1.2 in *P*. *atrox*, with the front limbs giving more robust slopes, and the hind limbs close to isometric growth. In every case, the least-squares regression slopes of the La Brea cats were in the same range of values as the slopes for living cats, suggesting that they have very similar growth trends.

Since there is no independent or dependent variable in this analysis, we calculated a reduced major axis instead of using the least-squares slope. First, we examined *P*. *atrox* (Fig 3), whose limbs might be expected to grow more gracile like those of a tiger or any other very large cat. The RMA slope for the humerus (Fig 3B) is 1.32, almost identical to the 1.36 slope of the tiger and most other living cats, and statistically gracile. The hind limb RMA slopes are 1.63 for the femur (Fig 3F) and 1.08 for the tibia (Fig 3H), again suggesting gracile to isometric growth in these limbs, and very similar to the gracile slopes seen in the living felids. Interestingly, the radius gives a slope of 0.34 (Fig 3D), which would indicate robust proportions. However, this is based on only eight specimens, the smallest sample in the entire study, so it might be affected by small sample size and inadequate range of sizes from smallest juveniles to adults. Likewise, the confidence interval for the RMA of most of the *P*. *atrox* is relatively large because of the small sample sizes, so the slopes of most of the limbs are not significantly different from isometric growth.

What about the slopes for *Smilodon*? Do they grow more robust, as predicted from previous research, or are they gracile like other cats? The humerus RMA slope (Fig 3A) is 1.65 and the radius RMA slope (Fig 3C) is 1.44, which are as gracile or more gracile than nearly all the other cats in the study, so they do not show a growth pattern of increasing robustness, contrary to expectations. There is no expectation that the hind limbs would be particularly robust, and indeed their RMA slopes are 1.65 for the femur (Fig 3E) and 1.71 for the tibia (Fig 3G), the most gracile of all the cats in this study. Due to the large sample sizes, nearly all the *Smilodon* slopes are statistically significant, and small confidence intervals show they are significantly different from the isometric slope, and even from other comparably sized cats like the tiger.

### Length vs. cross-sectional area

The results of the comparisons of length vs. cross-sectional area are shown in Tables 2, 4, 6 and 8, and Figs 3 and 4. The RMA slopes for both *S*. *fatalis* and *P*. *atrox* on all four limbs range from 0.40–0.77, which are very close to the 0.5 slope for isometric growth expected for a linear dimension vs. a squared dimension. The least squares results also give slopes in 0.4–0.7 range, consistent with the RMA slopes. This is a bit different from the isometric to gracile slopes of length vs. circumference, but clearly by this measure, they do not appear to be noticeably robust, nor is *S*. *fatalis* much different from *P*. *atrox*. Unfortunately, there are no published data for the length vs. area of limb bones in living cats to compare to, so the meaning of this result cannot be assessed comparatively.

## Discussion

As many paleontologists have long demonstrated, *Smilodon* forelimbs show many robust features of their shafts and articular surfaces (Fig 1), compared to other large cats of comparable size [1–10]. For example, the forelimb bones of *S*. *fatalis* are roughly the same length as those of the tiger, *P*. *tigris*, but the shafts of the humerus, ulna and radius are noticeably thicker in *S*. *fatalis* than in *P*. *tigris*, and they have broad articular surfaces with much heavier bony ridges and processes. As Meacham-Samuels and Van Valkenburgh [9] demonstrated, *S*. *fatalis* also has thickened cortical bone as compared to other similar-sized cats. However, we have neither the expertise nor the access to the appropriate equipment to exam the change in cortical thickness through growth. Based on the results of this study, however, we predict that the cortical thickness of forelimb bones in *Smilodon* will be relatively thicker than that of any other felid, even in the youngest kittens.

Thus, there is no question that based on adult bones, *S*. *fatalis* was much more robust in its forelimbs than other felids of comparable size. This is consistent with the idea that they were ambush predators rather than pursuit predators, and used their powerful forelimbs to quickly wrestle prey to the ground and pin it before slashing its vulnerable throat or belly with their saber-like canines.

However, there is no relative increase in robustness during growth of the forelimbs of *Smilodon* kittens compared to other felids (Figs 3 and 4, Tables 1–8). Instead, the dimensions of these bones follow the same trends as in other large cats, and nearly all their limbs (including the forelimbs) scale with the same gracility as other large cats.

If saber-tooth kittens grew with the same gracile trends as other large felids, how did they achieve their remarkably robust adult forelimbs? It is clear that the felid hallmark of increasing gracility of the limb bones during ontogeny was not changed by the need for robust limbs in adult *Smilodon*. Saber-tooth kitten growth does not override the canalized pattern of development of most felids to have long, gracile development of their limbs for running.

**Fig 5:**
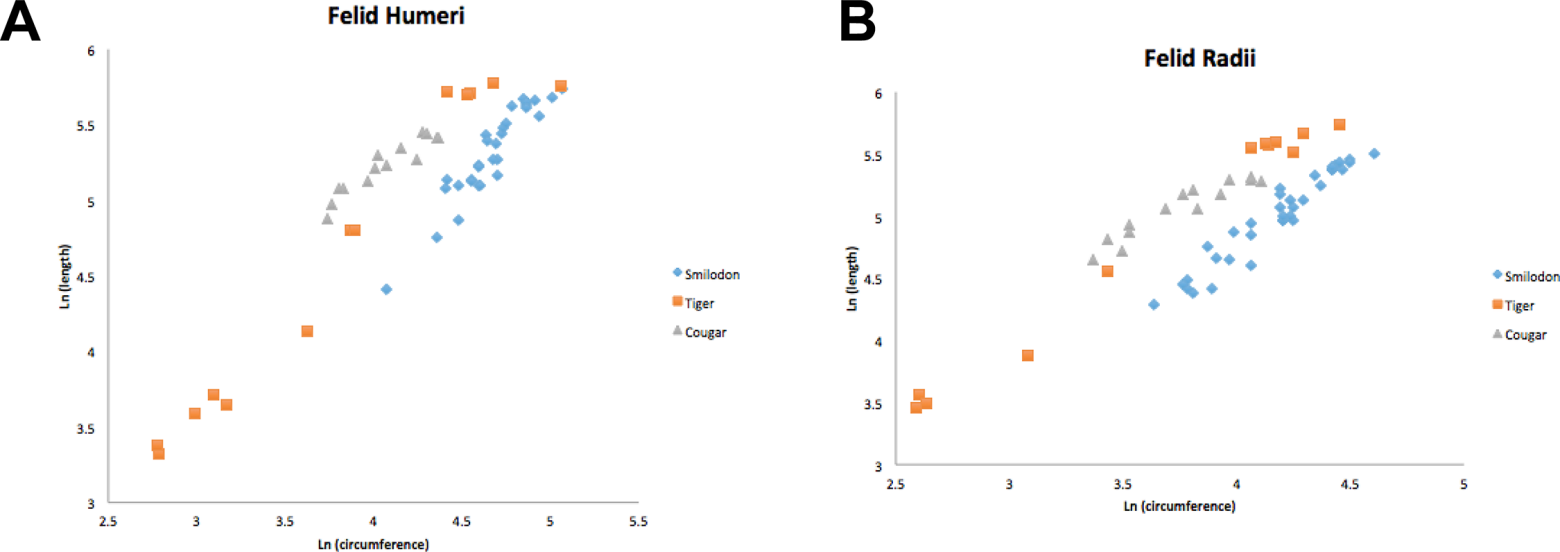
Comparative growth trends of kittens of saber-toothed cats (blue diamonds), tigers (orange squares) and cougars (gray triangles). In each case, the slope of their growth trend is the same, showing that all large felids grow along a common pattern emphasizing gracile forelimbs and cursoriality. However, the robustness of adult *Smilodon* forelimbs is achieved not by shifting the slope of the growth curve toward more robust directions in the axes, but by starting with more robust limbs as a kitten and maintaining the same growth trends as found in other felids. A. Humerus. B. Radius.

Instead, saber-tooth kittens achieved their adult limb proportions by starting out with relatively robust limbs when they were young kittens (Fig 5). In Fig 5, the growth curve of saber-tooth kittens has the same slope as those of tiger or cougar kittens, but it is shifted toward the more robust direction in the axes. For the same length, the saber-tooth kitten forelimb element (humerus or radius) always has a larger circumference than a comparably sized tiger or cougar. This is also indicated by the Y-intercept of the regression line of the humerus and radius, which was always higher in *Smilodon* than in the tiger (Tables 1 and 3). Thus, the unique features of robustness in *S*. *fatalis* forelimbs are achieved by starting with robust juvenile limb proportions, and they did not reprogram or override the primitive growth pattern of gracility inherent in felid ontogeny.

## Supporting information

S1 Table Original data for *Smilodon fatalis*. (XLSX)

S2 Table Original data for *Panthera atrox*. (XLSX)

S3 Table Results of reduced major axis analysis. (TXT)
